# Phenotypic and molecular consequences of overexpression of metal-homeostasis genes

**DOI:** 10.3389/fpls.2014.00080

**Published:** 2014-03-07

**Authors:** Danuta M. Antosiewicz, Anna Barabasz, Oskar Siemianowski

**Affiliations:** Faculty of Biology, Institute of Experimental Plant Biology and Biotechnology, University of WarsawWarszawa, Poland

**Keywords:** zinc (Zn), cadmium (Cd), nickel (Ni), hyperaccumulation, heavy metal, plant transformation, overexpression

## Abstract

Metal hyperaccumulating plants are able to store very large amounts of metals in their shoots. There are a number of reasons why it is important to be able to introduce metal hyperaccumulation traits into non-accumulating species (e.g., phytoremediation or biofortification in minerals) and to engineer a desired level of accumulation and distribution of metals. Metal homeostasis genes have therefore been used for these purposes. Engineered accumulation levels, however, have often been far from expected, and transgenic plants frequently display phenotypic features not related to the physiological function of the introduced gene. In this review, we focus on an aspect often neglected in research on plants expressing metal homeostasis genes: the specific regulation of endogenous metal homeostasis genes of the host plant in response to the transgene-induced imbalance of the metal status. These modifications constitute one of the major mechanisms involved in the generation of the plant's phenotype, including unexpected characteristics. Interestingly, activation of so-called “metal cross-homeostasis” has emerged as a factor of primary importance.

## Introduction

Engineered metal uptake, organ- and tissue-specific distribution, and tolerance in plants contribute to phytoremediation/phytoextraction (use of plants to remove metals from contaminated soils) and mineral biofortification (optimization of micronutrient contents in plant-derived food and exclusion of toxic metals). For both it is crucial to modify the metal concentration in plant parts (Palmgren et al., 2008; Verbruggen et al., 2009). For these purposes, plants have been transformed using metal homeostasis genes involved in uptake, compartmentalization, long-distance transport, and chelation of metals.

Contrary to expectations, the resulting plants expressing introduced genes have displayed characteristics unrelated to the physiological functions which these genes perform in their species of origin. For example, transformation with transporters not involved in metal uptake or with other metal homeostasis genes may lead to: (i)induction of an endogenous metal uptake system (e.g., Song et al., 2003; Thomas et al., 2003; Martinez et al., 2006; Gorinova et al., 2007; Korenkov et al., 2007); (ii)decreased uptake/accumulation, alteration of the status of a range of other metals, their distribution, and sensitivity to them (e.g., Harada et al., 2001; Li et al., 2004; Gasic and Korban, 2007; Wojas et al., 2008, 2009; Grispen et al., 2011; Barabasz et al., 2012, 2013 see also reviews by Kärenlampi et al., 2000; Pilon-Smits and Pilon, 2002; Eapen and D'Souza, 2005; Seth, 2012). The development of unexpected features is a manifestation of changes in a range of host-plant endogenous molecular and physiological pathways due to expression of the introduced gene/genes. As these alterations substantially contribute to generation of the plant's phenotype, understanding the underlying mechanisms is crucial for better planning of future modifications of metal accumulation and tolerance, and for Environmental Risk Assessment of genetically modified plants.

Increasing amounts of evidence indicate that molecular mechanisms governing the maintenance of metal homeostasis are not specific for each metal, but are interconnected by common pathways. This phenomenon, termed “cross-homeostasis,” results from regulation by several metals of the same transcription factor, the same metal transporter (which mediates—with different affinities—the translocation of more than one metal ion), or metal chelator (Sinclair and Krämer, 2012). In studies aimed at deciphering the mechanisms through which the metal-related phenotype of plants expressing a foreign gene is generated, specific regulation of the endogenous metal cross-homeostasis network of the host plant appeared to be a consequence of the expression of the transgene, and one of the major causes of the development of various characteristic features of transformants.

Only a few studies were performed so far to link modifications of an endogenous metal homeostasis network due to transgene expression with development of unforeseen characteristics of transgenic plants. This review will highlight the importance of understanding these processes for better engineering of metal-related features. Table 1 provides a summary of the studies discussed in the review.

**Table 1:**
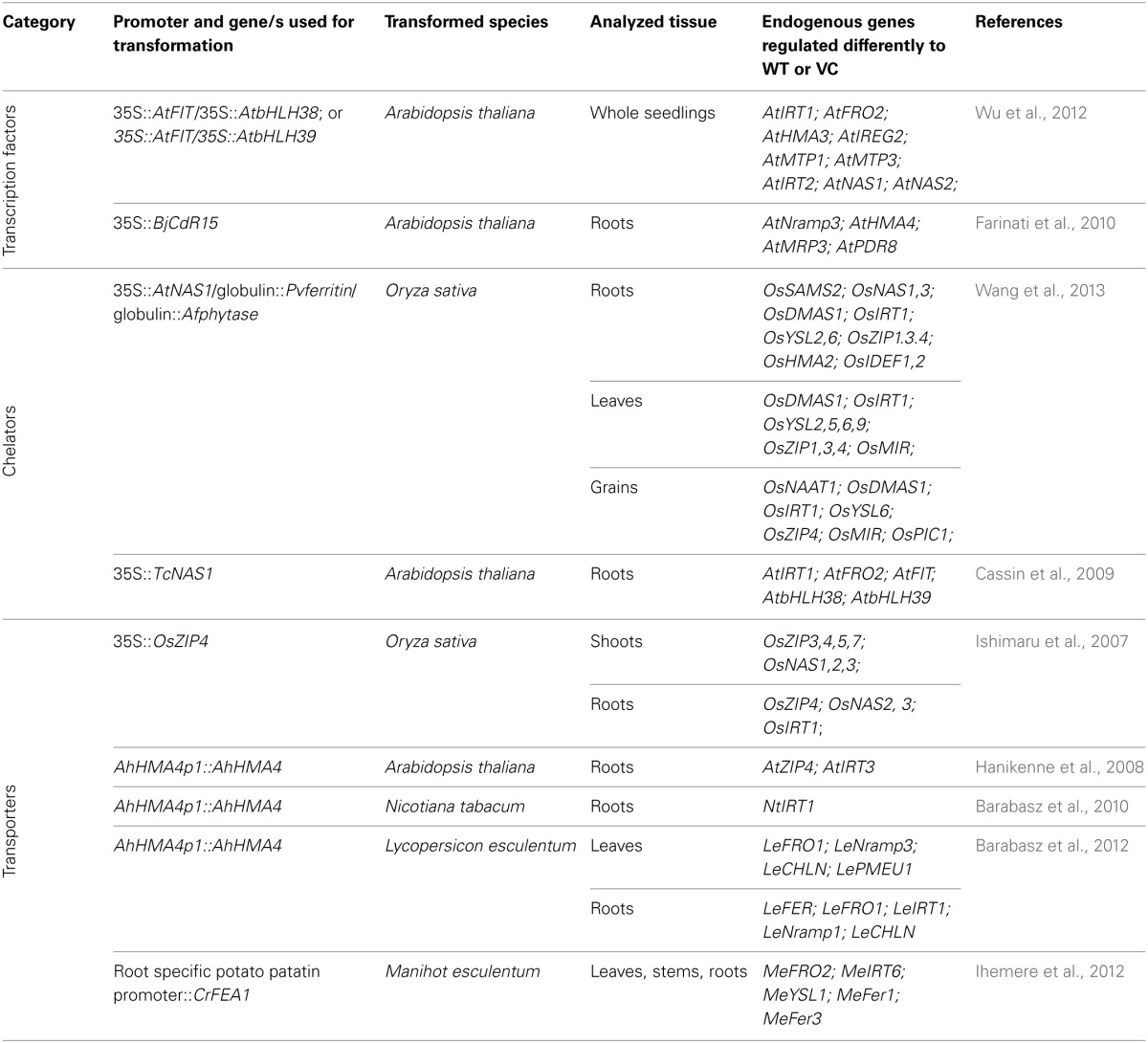
**List of transformations discussed in the review; shown are the genes and species used for transformation, and identified metal-homeostasis genes differentially expressed in transformants relative to wild-type (WT) or vector-controls (VC)**.

## Alteration of endogenous metal homeostasis mechanisms of plants expressing transgenes involved in metal homeostasis

### Expression of transcription factors

*IRT1* (ZRT-IRT-like Protein, ZIP family) mediates Fe^2+^ uptake by roots and, with a lower affinity, of Zn^2+^, Mn^2+^, Cd^2+^. In *Arabidopsis thaliana*, *AtIRT1* and *AtFRO2* (root surface ferric chelate reductase) are components of the Strategy I Fe-deficiency-induced Fe-acquisition system. Their expression is regulated by *FIT* (Fer-like Deficiency-Induced Transcription Factor), that forms heterodimers with one of the two transcription factors, *AtbHLH38* or *AtbHLH39* (Puig et al., 2007; Sinclair and Krämer, 2012).

Enhanced Cd tolerance in the model plant *Arabidopsis* was engineered by co-expression of transcription factors: 35S::*AtFIT* with 35S:: *AtbHLH38* (ox29/38) or 35S::*AtFIT* with 35S::*AtbHLH39* (ox29/39) (Wu et al., 2012). By studying the impact of transgene expression on the transcript level of chosen endogenous metal homeostasis genes, it was shown that their distinct regulation contributes to gaining new features related to a plant's response to Cd. In transformed plants, three fundamental changes that contribute to increased Cd tolerance were found at the molecular level.

First, transgenic plants had higher Fe levels in shoots (known to alleviate Cd-toxicity) resulting from greater Fe-uptake, associated with higher expression of Fe-deficiency inducible Fe-uptake genes *AtIRT1* and *AtFRO2*, and from more efficient upward translocation through unknown mechanisms.

Second, Cd concentrations were higher in roots, but lower in shoots. It was suggested that increased accumulation of Cd in roots resulted from detected constitutive enhancement of expression of genes known for their involvement in the retention of Cd in vacuoles and intracellular vesicles, such as *AtHMA3* (Heavy-metal ATPase) and Cd-regulated *AtIRT2* (Wu et al., 2012). Moreover, up-regulation of other Fe-deficiency response genes such as *AtIREG2* (Iron Regulated Gene, known to be co-regulated with *AtIRT1)* and *AtMTP3* (Metal Tolerance Protein) were noted. They contribute to metal homeostasis and their enhanced expression under low Fe status results from Fe-deficiency dependent enhancement of uptake of other divalent metal cations mediated by the Fe-uptake protein AtIRT1, which is a side effect of its low substrate specificity (Arrivault et al., 2006; Schaaf et al., 2006; Ricachenevsky et al., 2013).

Third, expression of *NAS1* and *NAS2* (nicotianamine synthase) was up-regulated and concentrations of metal-chelator nicotianamine (NA) were higher, indicating NA involvement in Cd-tolerance.

Cd tolerance and Cd translocation to shoots were also enhanced by overexpression of bZIP transcription factor *35S::BjCdR15* from *Brassica juncea* using the 35S promoter (Farinati et al., 2010) in *Arabidopsis*. It was shown that at longer exposure to Cd, the expression levels of *AtNramp3* (Natural resistance-associated macrophage protein)*, AtHMA4, AtMRP3* (Multidrug resistance-associated proteins from subfamily ABC) *and AtPDR8* (transporter from subfamily ABC) were significantly different in transgenic plants than in the wild-type. Interestingly, enhanced translocation of Cd to shoots of transformants was accompanied by lower expression of *AtHMA4* than in the wild-type. Since HMA4 controls Zn/Cd root-to-shoot translocation (Hussain et al., 2004; Wong and Cobbett, 2009), the detected enhancement of Cd concentrations in transgenic shoots is likely based on an *HMA4*-independent mechanism.

### Expression of chelator synthesis genes

Nicotianamine (NA), a non-proteinogenic amino acid, chelates metal ions, including Fe^3+^, Fe^2+^, Zn^2+^, Ni^2+^, Mn^2+^ (Curie et al., 2009). It is involved in processes regulating metal homeostasis and cross-homeostasis. Therefore, *NAS* genes were frequently used to engineer metal distribution and enhanced tolerance. The phenotypes of *NAS*-expressing plant species appeared to be very different with respect to alterations of Fe concentration/distribution, tolerance to low-to-high Fe levels, and responses to other metals (e.g., Ni, Zn, Mn), which indicated modifications of endogenous metal cross-homeostasis mechanisms.

The impact of transgene expression on endogenous metal homeostasis genes was investigated in two species, *A. thaliana* and *Oryza sativa*, which differ in Fe-uptake systems. As a dicot, *Arabidopsis* has reduction-based Strategy I. Grasses, e.g., rice or corn, use chelation-based Strategy II (roots release mugineic acid-MA, a Fe^3+^-chelating compound, subsequently taken up by YSL transporters). Rice, as an exception, also has the Strategy I system (Ishimaru et al., 2006). The *35S:*:*TcNAS* from *Thlaspi caerulescens* was overexpressed in *Arabidopsis* (Pianelli et al., 2005; Cassin et al., 2009). In rice, *35S:*:*AtNAS1* was expressed together with *Pvferritin* (Fe-storage protein) and *Afphytase* (catalyzing hydrolysis of phytic acid releasing chelated metals) under the rice seed storage globulin promoter (Wirth et al., 2009; Wang et al., 2013).

In both NA-overaccumulating transformants, distinct features were accompanied by different modifications of the expression of metal homeostasis genes, however, certain common changes emerged as well. In both transgenic plants enhanced expression of the Fe-deficiency induced Fe-uptake systems was reported, indicating increased Fe-deficiency status.

In *TcNAS*-expressing *Arabidopsis*, transcript level of *AtIRT1, AtFRO2* and three transcription factor genes (*AtFIT1*, *AtbHLH38*, and *AtbHLH39*) was higher than in the wild-type (Cassin et al., 2009). Overinduction of the Fe-uptake system increased the concentration of Zn and Mn in *NAS*-expressing *Arabidopsis*. Detected up-regulation of the Zn-transporter gene *AtZIP3* in these plants was interpreted as induction of endogenous metal transport mechanisms, to overcome the metal ion imbalances generated by expression of *TcNAS*.

In transgenic rice enhanced Fe uptake and translocation to grains (accumulating more Fe than wild-type) were noted (Wang et al., 2013). It was accompanied by induction of the Strategy I uptake system (as expression of *OsIRT1* was enhanced*)* but also by the Strategy II Fe-uptake system. Upregulation of genes from the MAs biosynthesis pathway were noted, including *OsSAMS2* (methionine synthetase); *OsNAAT1* (nicotianamine aminotransferase); *OsDMAS1* (deoxymugineic acid synthase), *OsNAS1*,*OsNAS2*,*OsNAS3*, and YSL transporters. Detailed tests performed at different developmental stages of transgenic and wild-type plants grown at high Fe and low Fe, shown also changes in expression of several metal homeostasis genes due to expression of *AtNAS1*/*Pvferritin*/*Afphytase* in rice. These include primarily *OsZIP1, OsZIP3, OsZIP4, OsZIP8, OsYSL2, OsYSL5, OsYSL6* encoding transporters which mediate translocation of different metals with different affinity.

Thus, ectopic expression of *TcNAS* in *Arabidopsis*, and *AtNAS1*/*Pvferritin*/*Afphytase* in rice, led to deregulation of homeostasis of several metals and induced endogenous metal-homeostasis molecular mechanisms to cope with these changes, thus contributing to the development of new plant's traits. Although the complete picture is not clear at the current stage of investigation, obtained results indicate that expression of *AtNAS1*/*Pvferritin*/*Afphytase* in rice contributes to better utilization of overproduced NA and DMA, and higher Fe storage capacity via ferritin, than the expression of only *TcNAS* in *Arabidopsis*. In the latter case, unwanted characteristics developed likely due to generation of more severe imbalances, primarily at the cellular level.

### Expression of metal transporters

#### ZIP4

*OsZIP4* from *Oryza sativa* is a Zn-deficiency-inducible gene expressed in the vasculature of roots and leaves. It encodes a plasma-membrane-localized protein responsible for Zn transport within plants, but not for its uptake from soil (Ishimaru et al., 2005). The overexpression of *35S::OsZIP4* in rice altered numerous endogenous pathways leading to 10-fold higher Zn concentrations in roots, 5-fold lower in shoots (accompanied by Zn-deficiency symptoms) and 50% reduction in grains (Ishimaru et al., 2007).

The comparison of transcription profiles of transgenic rice with vector-controls showed up-regulation of 236 genes in Zn-deficient shoots, of which 188 are Zn-deficiency-inducible genes. In contrast, out of 235 genes up-regulated in transgenic roots (accumulating high Zn) 102 genes are down-regulated by Zn-deficiency in vector control plants. Expression of 35S::*OsZIP4* deregulated also the expression of the endogenous *OsZIP4*. At control conditions, its transcript level was significantly lower in the roots and higher in the shoots, as compared with the vector control. Moreover, *35S::OsZIP4*-expression induced Fe-uptake and resulted in higher Fe concentrations in roots and shoots.

Thus, the transport activity of 35S::OsZIP4 in tissues other than those targeted in wild-types leads to substantial modification of Zn/Fe status at the cellular and tissue levels, stimulates uptake of Zn and Fe, and induces the Zn/Fe cross-homeostasis network to reconstitute the ion balance. Such modifications contribute to changes in Zn/Fe accumulation.

#### HMA4

It was shown that HMA4, a P1_B_-ATPase involved in Zn loading into xylem vessels, is responsible for control of Zn translocation to shoots both in non-accumulating *A. thaliana* and in the Zn-hyperaccumulator, *A. halleri* (Hussain et al., 2004; Hanikenne et al., 2008). Therefore *HMA4* was used for transformation to increase Zn levels in shoots of non-accumulating species. *AhHMA4* from *A. halleri* was expressed under its endogenous promoter in three plant species: *A. thaliana*, tobacco, and tomato (Hanikenne et al., 2008; Barabasz et al., 2010, 2012). Although the experimental conditions were very different (e.g., growth media, metals, time of exposure), the results provide new information about the modifications of endogenous molecular traits that contribute to the generation of the characteristic new features of genetically modifies plants.

The expression of *AhHMA4p_1_::AhHMA4* in *A. thaliana* introduced several traits of the Zn-hyperaccumulator (Hanikenne et al., 2008). First, loading of Zn into xylem vessels was enhanced. Second, the expression pattern of *AhHMA4p_1_::AhHMA4* in *A. thaliana* followed that characteristic of *A. halleri*. Moreover, similarly as in *A. halleri*, in transgenic *A. thaliana* roots the expression of Zn-deficiency-responsive genes *AtZIP4* and *AtIRT3* was enhanced. The increased expression of these genes in *A. halleri*, which is driven by Zn-deficiency generated by high activity of AhHMA4 contributes to the Zn-hyperaccumulation phenotype. Nevertheless, in *AhHMA4*-expressing *A. thalian*a exposed to 5 μM Zn, the shoot metal concentration was only slightly enhanced (1.16-fold). Increases in Zn concentrations in shoots to extents not characteristic of hyperaccumulators were also found in tobacco and tomato expressing *AhHMA4p_1_::AhHMA4* (Barabasz et al., 2010, 2012), but not at all doses in the range of tested Zn medium concentrations (from 0.5 to 500 μM Zn). Dose-dependence of metal root/shoot distribution, different in transgenic and wild-type, has also been reported for plants expressing *35S::AtHMA4* as well as other metal transporters such as *CAXs, AtECA3* and *HvHMA2* (Korenkov et al., 2007; Barabasz et al., 2011, 2013; Siemianowski et al., 2011). The Zn-supply dependent modifications of Zn concentrations in the shoots of plants expressing *HMA2, HMA4*, and *ECA3* were listed in the Table 2. It has previously been reported for *A. thaliana*, *A. halleri*, and *T. caerulescens* that distinct homeostatic mechanisms underlie low, sufficient, and excess Zn statuses (Talke et al., 2006; van de Mortel et al., 2006). Therefore, phenomenon of the metal-supply dependent metal-accumulation pattern detected in transgenic plants was thought to result from different molecular backgrounds at varying metal statuses, against which the expression of a transgene takes place.

**Table 2:** **Zn-supply dependent modifications of Zn concentration in the shoots of plants expressing genes listed in the first column (relative to wild-type)**.

**Promoter/gene used for transformation**	**Transformed species**	**Alterations of Zn concentration in shoots of transgenic plants (relative to wild-type) exposed to 0.5; 1; 10; 100; 150; and 200 μM Zn**						**References**
		**0.5 μM Zn**	**1 μM Zn**	**10 μM Zn**	**100 μM Zn**	**150 μM Zn**	**200 μM Zn**	
35S::*AtHMA4*	Tobacco	ND	–	H	ND	–	ND	Siemianowski et al., 2011
35S::*AtHMA4*-Cterm	Tobacco	H	–	H	L	–	L	
35S::*AtHMA4*-trunc	Tobacco	ND	–	ND	L	–	L	
*AhHMA4p1::AhHMA4*	Tobacco	H^*^	L	ND	–	ND	ND	Barabasz et al., 2010
*AhHMA4p1::AhHMA4*	Tomato	–	ND	H	–	–	–	Barabasz et al., 2012
35S::*HvHMA2*	Tobacco	–	ND	H	ND	–	–	Barabasz et al., 2013
35S::*AtECA3*	Tobacco	ND	–	L	–	–	–	Barabasz et al., 2011

Changes in Zn concentrations in shoots of transgenic tobacco or tomato (relative to wild-type) due to expression of a given gene, grown in the presence of 0.5; 1; 10; 100; 150; and 200 μM Zn, were marked as: ND, no difference between transgenic and wild-type plant; H, higher in transgenic plants than in wild-type; L, lower in transgenic plants than in wild-type; –, not examined;

* , only in upper leaves.

At varying metal levels in the medium, the expression pattern of endogenous metal homeostasis genes in transgenic plants also differed from wild-type. For example, in tobacco transformed with *AhHMA4p_1_::AhHMA4*, the expression of *NtIRT1* was enhanced specifically upon Zn-deficiency and at 10 μM Zn when compared with control conditions or with the *NtIRT1* transcript level in the wild-type (Barabasz et al., 2010). This suggests that the transcript abundance of the Fe/Zn uptake gene *NtIRT1* undergoes differential regulation in transformants over a range of Zn exposures. This might result from distinct Zn/Fe-statuses under these conditions in connection with differential modification of the endogenous metal homeostasis network resulting from expression of the transgene. Similarly, in tomato expressing *AhHMA4p1::AhHMA4* there was a substantial difference in the expression levels of *LeFER, LeIRT1, LeNramp1, LeCHLN (LeNAS)*, and *LePMEU1* (pectinmethylesterase) between transgenic and wild-type plants exposed to low and high Zn (Barabasz et al., 2012).

It was found that the expression of *HMA4* in tomato, tobacco, and *Arabidopsis* makes plants more sensitive to Zn (Hanikenne et al., 2008; Barabasz et al., 2010, 2012; Siemianowski et al., 2011). Expression of the Zn export gene *35S::AtHMA4* in tobacco and *AhHMA4p1::AhHMA4* in tomato led to overloading of the apoplast with Zn (Barabasz et al., 2012, Siemianowski et al., 2013). In *35S::AtHMA4*-expressing tobacco enhanced Zn concentrations in the apoplast were shown to be critical for development of leaf necrosis—symptoms of Zn-sensitivity (Siemianowski et al., 2013). Molecular consequences of *HMA4* expression were also detected within the cell wall. In *AhHMA4p1::AhHMA4*-expressing tomato, a 20-fold higher Zn concentration in the apoplast was associated with up-regulation of *LePMEU1* (Barabasz et al., 2012), the cell-wall remodeling pectinmethylesterase (Pelloux et al., 2007). These indicate that transgene-induced processes leading to cell-wall modifications might significantly contribute to the response of transgenic plants to metals. A role for pectinmethylesterase in the apoplastic storage of Zn or Hg had been already suggested (Heidenreich et al., 2001; Hassinen et al., 2007). Moreover, studies performed on *AhHMA4p1::AhHMA4*-expressing tomato showed that higher than wild-type sensitivity to excess Zn was accompanied by: (i) decreased Fe concentrations in leaves; (ii) enhanced expression of Fe-deficiency-induced *LeFRO1*, *LeIRT1*, and *LeFER* (a functional ortholog of *AtFIT*)*;* (iii) higher activity of FRO; (iv) higher transcript abundance of *LeNramp1*, which is a transporter involved in redistribution of iron from vacuolar stores under Fe-limiting conditions (Bereczky et al., 2003); (v) higher *LeCHLN* expression. Thus, disturbances in apoplast/symplast Zn-status due to expression of Zn-export genes (e.g., *HMA4*) seem to be a key factor inducing ion imbalances at the cellular/tissue level. Subsequent modifications of transcription profiles of metal homeostasis genes in engineered plants underlie development of characteristic features unrelated to the physiological functions of introduced genes.

Another detected unforeseen characteristic feature resulting from the ectopic expression of a transgene, was decreased Cd uptake/accumulation in roots and shoots of tobacco (*Nicotiana tabacum* v.Xanthi) expressing *35S::AtHMA4* (Siemianowski et al., 2011). For understanding the mechanisms underlying this Cd-dependent phenotype and to help predict the consequences of a transgene expression for potential phytoremediation/biofortification-based strategies, microarray analysis was performed to identify metal homeostasis genes that were differentially expressed in roots of Cd-exposed *AtHMA4*-expressing tobacco relative to the wild type (Siemianowski et al., 2014). It was shown that restriction of Cd uptake/accumulation in *AtHMA4*-expressing plants was not related to downregulation of genes involved in Cd uptake. The expression level of *NtIRT1* and *NtZIP1* were higher in transgenic plants indicating generated Fe- and Zn-deficiency status due to *AtHMA4* expression. Interestingly, due to ectopic expression of 35S::*AtHMA4* the physical apoplastic barrier within the external cell layer developed, which was considered responsible for the reduction of Cd uptake/accumulation. Biochemical and microscopic analysis of roots showed that expression of *AtHMA4* caused an induction of cell wall lignification in the external cell layers that was accompanied by enhanced H_2_O_2_ accumulation. These changes were accompanied by the upregulation of genes involved in cell wall lignification (*NtHCT*, *NtOMET, NtPrx11a)*.

## Implications

A challenge in engineering metal tolerance, accumulation and distribution in plants is to eliminate unwanted, unfavorable features. The observed phenotypes are unexpected based solely on the molecular function of the protein encoded by the transgene (for example Zn transport), but result from a complex interaction between this molecular function, the expression pattern and strength of the transgene and the host response to the modification of the metal homeostasis network.

Recent studies indicate that deregulation of a metal balance due to activity of a protein encoded by a gene used for transformation (primarily in cells/tissues other than in the species from which the gene was cloned) contribute to generation of plant's characteristics. Changed metal status at a cellular/tissue/organ level activates endogenous metal homeostasis mechanisms to combat the generated imbalance. Thus, the secondary effects induced by expression of introduced genes are, in fact, an integral part of the mechanisms underlying development of unwanted features, although usually they have remained unstudied.

Ectopic expression under a strong constitutive promoter (e.g., CaMV35S) disturbs status of metal/metals, consequently metal homeostasis networks, everywhere in a plant (Siemianowski et al., 2013). Localized transgene expression under tissue-/organ-specific promoters should result in modifications only in targeted tissues/organs, however, we must know where exactly in a host plant its expression takes place or where proteins accumulate. In the majority of studies this was only assumed, without direct demonstration. This is a challenge for future studies.

Another difficulty in engineering a metal-related trait results from the metal cross-homeostasis phenomenon. It is becoming evident that changes in the status of one metal due to the expression of a chosen gene/genes generate multifactorial responses related to other metals as part of the regulation of an endogenous cross-homeostasis network. In targeted expression, activation of cross-homeostasis mechanisms still takes place also in non-targeted organs leading to effects poorly recognized. Therefore, in devising strategies for engineering a specific metal-related feature, it is necessary to keep in mind that broad spectrums of metals and processes should be investigated. For that purpose, identification and use of marker genes (ideally regulated exclusively or primarily by one metal) could be of help. Current knowledge is very limited, but available data indicate that the Strategy I Fe uptake system is affected in plants transformed with different genes involved in the metabolism of Zn, Fe and other metals, likely as a result of modified cellular/tissue/organs metal-status.

The complexity of difficulties in engineering metal accumulation/distribution is also manifested by the phenomenon of metal-supply-dependent metal distribution, in transgenic plants (phenomenon known for the wild-type). Contribution of the interplay between transgene expression/protein activity and the host plant metal homeostasis network was suggested. It is, therefore, necessary to use a broad range of metal concentrations for tests, otherwise modifications resulting from transgene expression could be overlooked. This also points to difficulties in engineering a plant displaying the desired metal-related trait (e.g., hyperaccumulation) under varying conditions of metal supply.

Moreover, it is worth noting that molecular analysis of modifications of endogenous metal homeostasis pathways altered in transgenic plants can provide knowledge not only about mechanisms generating the phenotype, but also about the regulation of cross-homeostasis mechanisms by identification of sets of genes playing a key role in this phenomenon.

### Conflict of interest statement

The authors declare that the research was conducted in the absence of any commercial or financial relationships that could be construed as a potential conflict of interest.
